# Reprogramming the Epigenome With Vitamin C

**DOI:** 10.3389/fcell.2019.00128

**Published:** 2019-07-16

**Authors:** Taylor Lee Chong, Emily L. Ahearn, Luisa Cimmino

**Affiliations:** ^1^Department of Biochemistry and Molecular Biology, Miller School of Medicine, University of Miami, Miami, FL, United States; ^2^Sylvester Comprehensive Cancer Center, Miller School of Medicine, University of Miami, Miami, FL, United States

**Keywords:** vitamin C, stem cell reprogramming, TET, Jumonji C, cancer

## Abstract

The erasure of epigenetic modifications across the genome of somatic cells is an essential requirement during their reprogramming into induced pluripotent stem cells (iPSCs). Vitamin C plays a pivotal role in remodeling the epigenome by enhancing the activity of Jumonji-C domain-containing histone demethylases (JHDMs) and the ten-eleven translocation (TET) proteins. By maintaining differentiation plasticity in culture, vitamin C also improves the quality of tissue specific stem cells derived from iPSCs that are highly sought after for use in regenerative medicine. The ability of vitamin C to potentiate the activity of histone and DNA demethylating enzymes also has clinical application in the treatment of cancer. Vitamin C deficiency has been widely reported in cancer patients and has recently been shown to accelerate cancer progression in disease models. Therapies involving high-dose vitamin C administration are currently gaining traction in the treatment of epigenetic dysregulation, by targeting aberrant histone and DNA methylation patterns associated with cancer progression.

## Introduction

Vitamin C is an essential micronutrient for humans with important roles as a systemic antioxidant. Unlike most other mammals, including many primates and mice, humans are incapable of synthesizing their own vitamin C due to a loss of function mutation in the L-gulono-γ-lactone oxidase (GULO) gene that is required to catalyze the final step of vitamin C formation in the liver (Drouin et al., 2011). In addition to its role as a cellular antioxidant, vitamin C is a critical cofactor of Fe^2++^ and α-ketoglutarate-dependent dioxygenases (α-KGDDs) maintaining and enhancing the activity of these enzymes (Young et al., 2015). Included amongst the diverse group of α-KGDDs regulated by vitamin C are erasers of epigenetic modifications, such as the Jumonji-C domain-containing histone demethylases (JHDMs) and the ten-eleven translocation (TET) family of DNA hydroxylases. Numerous studies over the last decade have revealed that the simple addition of vitamin C to the culture media of somatic cells during reprogramming improves the efficiency and quality of induced pluripotent stem cell (iPSC) formation (Esteban et al., 2010; Wang et al., 2011; Stadtfeld et al., 2012). Vitamin C, by enhancing the catalytic activity of JHDMs and TETs drive histone and DNA demethylation in somatic cells that allow pluripotency genes to turn on while simultaneously erasing the epigenetic memory of the adult cell state. In addition to the epigenetic changes driven by JHDMs and TETs, vitamin C can also target other α-KGDDs that regulate metabolism, DNA repair and DNA/RNA de-methylation that may play important roles in fine-tuning the reprogramming stages of somatic cells.

The potential to reprogram somatic cells from any adult cell of origin into pluripotent stem cells, that regain their ability to generate embryos and develop into alternative mature lineages, has provided an invaluable tool in the study of development, disease, and for generating tissues with novel therapeutic applications in regenerative medicine (Takahashi and Yamanaka, 2006; Zhao et al., 2009). Vitamin C-mediated remodeling of the epigenome also has important implications in the treatment of cancer. Epigenetic dysregulation is a hallmark of cancer initiation and progression, causing aberrant gene expression patterns and genomic instability (Garnis et al., 2004). Epigenetic erasers such as JHDMs and TETs are frequently mutated in cancer, resulting in histone and DNA hypermethylation phenotypes that block tumor cells from responding to differentiation cues and provide protection in response to chemotherapy. Environmental stimuli can also play a major role in the epigenetic plasticity of disease states and alter the course of disease progression independently of mutations in epigenetic regulators (Flavahan et al., 2017). Vitamin C treatment at high-doses is now being explored as a novel therapeutic approach to overcome metabolic or epigenetic dysregulation and reprogram the cancer epigenome, allowing cells to regain their ability to differentiate and improve their responsiveness to standard chemotherapies.

## Somatic Cell Reprogramming With Vitamin C

The enforced expression of a defined set of transcription factors such as Oct4, Sox2, Klf4, and c-Myc (OSKM) is sufficient to generate iPSCs from mouse and human somatic cells that replicate the naïve “ground-state” of blastocyst-derived ESCs (Takahashi and Yamanaka, 2006; Takahashi et al., 2007; Yu et al., 2007). However, reprogramming efficiency is often very low and influenced by multiple factors including donor cell age, passages in culture, lineage of origin, or stage of development (Eminli et al., 2009; Li et al., 2009; Marion et al., 2009). The importance of erasing epigenetic memory in iPSCs is evidenced by the tendency for reprogrammed cells to exhibit a differentiation bias toward the donor cell lineage, that may restrict their capacity for widespread use in disease modeling or treatment (Kim et al., 2010; Polo et al., 2010).

The process of becoming an iPSC is slow, and the forced expression of OSKM factors increases the levels of reactive oxygen species (ROS) that can cause DNA damage and senescence (Banito et al., 2009). Vitamin C was originally added to the culture media of reprogramming mouse and human cells for its antioxidant properties, in an attempt to mitigate the effects of ROS that could potentially hamper the efficiency and quality of reprogramming (Esteban et al., 2010). However, in comparison to other antioxidants such as glutathione, N-acetylcysteine, vitamin E and lipoic acid, vitamin C was found to be substantially more efficient at enhancing proliferation of mouse ESCs and iPSC generation from mouse or human fibroblasts (Esteban et al., 2010). Based on the role of vitamin C as a cofactor for α-KGDDs, such as JHDMs, it was postulated that the mechanism by which vitamin C could facilitate reprogramming was through increased histone demethylation, given that histone demethylases were known to be important for the expression of the ESC master transcription factor Nanog (Cloos et al., 2008). Co-culture with inhibitors of the vitamin C-dependent α-KGDDs, such as the iron chelator desferrioxamine (DFO) or the α-KG analog dimethyloxalylglycine (DMOG), led to impaired iPSC formation from mouse embryonic fibroblasts (MEFs; Wang et al., 2011), formally implicating these enzymes in the mechanism of vitamin C-mediated somatic cell reprogramming. Subsequent studies also showed that vitamin C increased the rate of human ESC proliferation, and promoted DNA demethylation at genomic loci known to undergo widespread loss of methylation during the reprogramming of somatic cells into iPSCs (Chung et al., 2010). Vitamin C was also shown to prevent DNA hypermethylation and maintain expression of the imprinted *Dlk1-Dio3* gene cluster, where loss of imprinting at this locus was known to cause the abnormal development of mice generated from iPSCs (Stadtfeld et al., 2010, 2012). The ability of vitamin C to increase the efficiency of reprogramming and improve the quality of iPSCs was therefore attributed to its ability to modulate the epigenome.

## Targeting Histone Demethylases During Reprogramming With Vitamin C

Vitamin C is required for the optimal activity and demethylation capacity of several JHDMs (Tsukada et al., 2006), which include over 20 α-KGDDs in humans that hydroxylate and remove methyl groups from lysines in histones (Klose et al., 2006). Histone demethylation is catalyzed by the Jumonji C (JmjC)-catalytic domain to produce a highly reactive oxoferryl species that hydroxylates the methylated substrate, allowing spontaneous loss of the methyl group as formaldehyde (Clifton et al., 2006). The most well studied JHDMs target methylated lysines in the tail of histone H3. JHDM1A/B (KDM2A/B) specifically demethylate H3K36; JMJD1/JHDM2A (KDM3A) demethylates H3K9; JHDM3A/JMJD2A-E (KDM4A-E) demethylate both trimethylated H3K36 and/or H3K9, and UTX/JMJD3 (KDM6A/B) demethylate H3K27, all of which can be modulated by vitamin C to regulate chromatin state and gene expression (Klose et al., 2006; Cloos et al., 2008; Pedersen and Helin, 2010; Monfort and Wutz, 2013; Burchfield et al., 2015).

An important barrier to somatic cell reprogramming is methylation of H3K9 that marks facultative and constitutive heterochromatin (Chen J. et al., 2013). By increasing Jmjd1a/b (Kdm3a/b) and Jmjd2b/c (Kdm4b/c) activity in mouse ESCs and during mouse pre-iPSC-to-iPSC transition, vitamin C induces a specific loss of H3K9me2/me3 at core pluripotency gene loci (Chen J. et al., 2013; Ebata et al., 2017). Utx (Kdm6a) is another JmjC-domain-containing α-KGDD that demethylates H3K27me3 and is a crucial regulator of pluripotency induction during mouse and human somatic cell reprogramming (Mansour et al., 2012). Vitamin C-treated mouse ESCs in culture change their distribution of H3K27me3 across the genome (Ebata et al., 2017), however, the effect on total levels were shown to be minor suggesting that modulation of Utx by vitamin C during reprogramming may be qualitative (locus-specific) rather than quantitative and requires further study. Vitamin C has also been shown to increase the activity of Jhdm1a/1b (Kdm2a/b) to promote H3K36me2/3 demethylation in MEFs in culture and during iPSC reprogramming (Wang et al., 2011). The localization of H3K36me2/me3 within gene bodies is important for maintaining active gene transcription, the removal of which facilitates silencing of the *Cdkn2a* (*Ink4/Arf*) locus, a critical regulator of senescence that causes growth arrest in cells, and prevents efficient reprogramming (He et al., 2008; Li et al., 2009; Tzatsos et al., 2009). Aged cells exhibit higher levels of Ink4/Arf, limiting their efficiency and fidelity of reprogramming (Li et al., 2009). Active silencing of the *Ink4/Arf* locus in the presence of vitamin C could also help iPSCs derived from aged donor cells to reprogram more effectively.

## Targeting TET DNA Demethylases With Vitamin C During Somatic Cell Reprogramming

**FIGURE 1:**
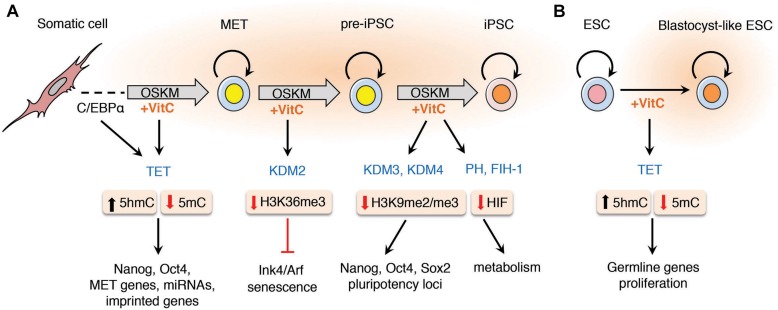
Vitamin C promotes somatic cell reprogramming by enhancing the activity of α-KGDDs. The addition of vitamin C to the culture medium of somatic cells during reprogramming enhances the activity of α-ketoglutarate dependent dioxygenases (α-KGDDs) including Jumonji-C domain-containing histone demethylases (JHDMs/KDMs), ten-eleven translocation (TET) proteins, prolyl hydroxylases (PH) and the asparaginyl hydroxylase FIH-1. **(A)** During somatic cell reprogramming it has been shown that transient C/EBPα expression up-regulates Tet2 expression that primes cells for mesenchymal-to-epithelial transition (MET). MET is an essential step required for the initiation of reprogramming that is dependent on TET-mediated DNA hydroxylation and demethylation of the enhancers and promoters of pluripotency genes (e.g., Nanog and Oct4) and for the expression of MET genes, miRNAs and to prevent hypermethylation and silencing of imprinted loci. The hypomethylation of histones by JHDMs such as KDM2 targets H3K36me3 for demethylation that suppresses the expression of senescence-inducing factors Ink4/Arf. Vitamin C also increases loss of H3K9me2/me3 by enhancing KDM3/4 activity to maintain expression at pluripotency loci during the final stages of reprogramming pre-iPSCs in to fully pluripotent iPSCs. Vitamin C may also reduce oxidative stress and increase the activity of hypoxia-inducible factor (HIF) prolyl hydroxylases and FIH-1 that promote the degradation of HIF that has also been shown to be important in the final stage of reprogramming. **(B)** Vitamin C treatment increases the proliferation of embryonic stem cells (ESCs) in culture and promotes demethylation of germline genes by enhancing TET activity to replicate the naïve “ground-state” of blastocyst-derived ESCs.

The TET proteins (TET1-3) are a sub-family of α-KGDDs that catalyze the hydroxylation of 5-methylcytosine (5mC) residues in DNA to generate 5-hydroxymethylcytosine (5hmC) (Tahiliani et al., 2009), and through successive oxidation reactions, 5-formylcytosine (5fC) and 5-carboxylcytosine (5CaC). Vitamin C treatment has been shown to dramatically increase 5hmC, 5fC, and 5caC production in ESCs and enhance the reprogramming of mouse and human fibroblasts to iPSCs in a TET-dependent manner (Blaschke et al., 2013; Chen Q. et al., 2013; Minor et al., 2013; Yin et al., 2013). The oxidative products of 5mC catalyzed by TET proteins can be stable modifications in the genome or transient modifications that provide a trigger for active or passive DNA demethylation (Tahiliani et al., 2009; Ito et al., 2010; Zhang et al., 2010). Several studies have shown that TET expression is essential for reprogramming. *Tet1-* or *Tet2*-depleted MEFs are unable to generate OSKM-mediated iPSCs (Doege et al., 2012; Costa et al., 2013), and Tet1-3 triple knockout (TKO) MEFs are blocked during mesenchymal-to-epithelial transition (MET), a crucial initial step in the reprogramming of MEFs into iPSCs (Hu et al., 2014). The expression of OSKM in MEFs has been shown to activate *Tet2* expression, leading to a genome-wide increase in 5hmC and DNA demethylation at the loci of pluripotency factors such as *Nanog* and *Esrrb* that correlates with their enhanced expression (Doege et al., 2012). The transient expression of C/EBPα in mouse primary B cells sensitizes them for OSKM-mediated reprogramming by up-regulating *Tet2* expression and facilitating accessibility of pluripotency genes to Oct4 binding that leads to significantly enhanced iPSC generation (Di Stefano et al., 2014) (Figure 1A). Increased Tet2 activity induced by induced by C/EBPα in reprogramming B cells promotes 5hmC formation and DNA demethylation at enhancers and promoters of pluripotency genes and Tet2 is physically recruited to the chromatin of reprogramming cells via interaction with C/EBPα, Klf4 and the naive pluripotency factor Tfcp2l1 (Sardina et al., 2018). TET1 and TET2 proteins also physically interact with NANOG, and their overexpression promotes hydroxylation and DNA demethylation at the *Oct4* locus resulting in increased reprogramming efficiency (Costa et al., 2013; Gao et al., 2013). In the absence of all three TET proteins, Tet TKO MEFs fail to demethylate and reactivate the miR-200 family of genes, resulting in the MET block that renders these cells unresponsive to reprogramming (Hu et al., 2014).

TET proteins are also important for the maintenance of ESC pluripotency in response to vitamin C. ESCs treated with vitamin C in culture accumulate 5hmC at transcriptional start sites of genes known to be enriched for TET binding (Williams et al., 2011; Wu and Zhang, 2011; Xu Y. et al., 2011; Chen Q. et al., 2013; Deplus et al., 2013; Vella et al., 2013) that is followed by DNA demethylation of germ-line genes normally expressed during formation of a blastocyst-like state (Blaschke et al., 2013; Yin et al., 2013) (Figure 1B). DNA hypermethylation at imprinted loci has been reported in the progeny of mice deficient in *Tet1* and *Tet2* (Dawlaty et al., 2013), and vitamin C maintains active histone marks (H3K4me2/3) and prevents the DNA hypermethylation and silencing of imprinted genes at the *Dlk1-Dio3* gene cluster (Stadtfeld et al., 2012). Vitamin C can also work synergistically with other factors, such as Vitamin A (retinoic acid, RA), to enhance mouse iPSC reprogramming (Esteban et al., 2010; Schwarz et al., 2014; Hore et al., 2016). RA has no direct effect on TET enzymatic activity but can increase TET expression levels, leading to increased 5hmC and increased DNA demethylation (Hore et al., 2016). When supplemented in combination, vitamin C enhances the activity of an increased pool of TET protein driven by RA signaling, resulting in greater reprogramming efficiency of primed mouse ESCs to naïve pluripotency (Hore et al., 2016). Vitamin C can also facilitate rapid and synchronous reprogramming of MEFs and other mouse cell types, such as progenitor B and myeloid cells, in combination with small molecule inhibitors of GSK3β and TGFβ that enhance iPSC formation by an order of magnitude greater than either treatment alone (Bar-Nur et al., 2014; Vidal et al., 2014).

## Additional Roles for Vitamin C in Reprogramming Via α-Kgdds

Vitamin C enhances the activity of another subset of α-KGDDs, the hypoxia inducible factor (HIF) prolyl hydroxylases (PHs) that regulate the proteasomal degradation of HIF proteins (Keith and Simon, 2007). HIFs are oxygen-sensing transcription factors made up of α and β subunits that dimerize and translocate to the nucleus under conditions of hypoxia to regulate the expression of genes involved in oxygen homeostasis, glucose metabolism, angiogenesis, erythropoiesis and iron metabolism (Semenza, 1999; Mohyeldin et al., 2010). Hypoxia is a key feature of the stem cell niche, known to increase the self-renewal capacity of ESCs, adult stem cells and enhance the generation of iPSCs (Yoshida et al., 2009; Mohyeldin et al., 2010). During reprogramming, HIF proteins are required to initiate the metabolic switch from oxidative to glycolytic metabolism, an essential step for the initial stages of somatic cell reprogramming (Folmes et al., 2011; Panopoulos et al., 2012). During hypoxia, the HIFα subunits (HIF1α and HIF2α) are stabilized and help drive the metabolic switch to glycolysis. However, prolonged stabilization of HIF-2α in the final stages of reprogramming will cause a significant block in the acquisition of a fully pluripotent ESC-like state (Zhou et al., 2012; Mathieu et al., 2014). Vitamin C may therefore have stage-specific roles in fine-tuning the process of reprogramming. By targeting HIF-2α for degradation, vitamin C-mediated enhancement of HIF PHs may help pre-IPSCs convert into fully reprogrammed iPSCs at this crucial final step in the acquisition of pluripotency (Takahashi and Yamanaka, 2006) (Figure 1A). Vitamin C also enhances the activity of the asparaginyl hydroxylase factor inhibiting HIF-1 (FIH-1), an important suppressor of the transcriptional activity of HIF (Lando et al., 2002), consistent with studies showing a vitamin C-mediated reduction in the mRNA expression levels of HIF genes in leukemia cell lines (Kawada et al., 2013).

The alkylated DNA repair protein AlkB homologs (ALKBHs) are another group of α-KGDDs with the potential to influence somatic cell reprogramming in response to vitamin C treatment. ALKBH1, a histone dioxygenase that removes methyl groups from histone H2A, is more highly expressed in stem cells than differentiated cells and its expression increases during iPSC reprogramming (Guenther et al., 2010; Ma et al., 2012). ALKBH1 interacts directly with the core pluripotency factors Oct4, Sox2, and Nanog at overlapping sites on chromatin and influences the expression of miRNAs important for maintaining ESC self-renewal and pluripotency (Ougland et al., 2012). Other ALKBHs include two RNA demethylases, the fat-mass and obesity-associated (*FTO*) gene and AlkB homolog 5 (*ALKBH5*), that catalyze the removal of m^6^A modifications in mRNA transcripts (Jia et al., 2011; Zheng et al., 2013). Increased m^6^A abundance promotes the reprogramming of MEFs to pluripotent stem cells; conversely, reduced m^6^A levels impede reprogramming (Chen et al., 2015). Vitamin C has recently been shown to promote erasure of m^6^A during the differentiation of pig oocytes (Yu et al., 2018), however it is not yet clear how these enzymes are affected by vitamin C treatment during somatic cell reprogramming.

## Advancing Stem Cell Therapies With Vitamin C

The reprogramming of adult cells into iPSCs has provided researchers with the opportunity to generate a renewable source of cells for application in a range of stem cell therapies. Stem cells are highly sought after in regenerative medicine for the treatment of injury, aging, neurodegenerative diseases, heart failure, skin or eye disorders and cancer (Wu and Hochedlinger, 2011; Scudellari, 2016). Patient-derived iPSC-based disease models can be used as a source of genetically matched tissues for autologous cell-replacement therapy and as an *in vitro* platform for high-throughput screening of treatment modalities or for novel drug target discovery. In the first clinical trial using iPSCs derived from skin cells, sheets of retinal pigment epithelium were generated for the treatment of patients with age-related macular degeneration with reports of improved vision and a block in further degeneration (Mandai et al., 2017). However, important concerns were raised related to genetic changes in patient-derived iPSCs that halted further trials in these patients.

**FIGURE 2:**
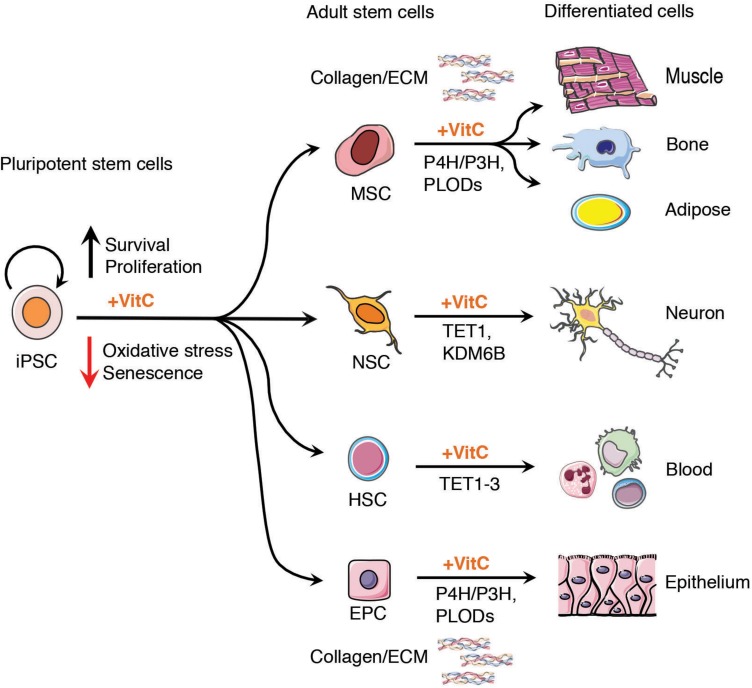
Vitamin C enhances stem cell therapeutic potential. Vitamin C maintains the proliferation and self-renewal capacity of tissue specific stem and progenitor cells derived from iPSCs in culture, including epithelial stem and progenitor cells (EPC), neural stem cells (NSC), mesenchymal stem cells (MSCs), and hematopoietic stem cells (HSCs). The addition of vitamin C to the culture medium of cells enhances the activity of α-ketoglutarate dependent dioxygenases (α-KGDDs) including Jumonji-C domain-containing histone demethylase (JHDM) such as KDM6B, ten-eleven translocation (TET1-3) proteins, prolyl Hydroxylases (P4H, P3H) and pro-collagen-lysine α-KG 5-dioxygenases (PLODs). Vitamin C may serve as a key adjuvant in preclinical models of iPSC-based regenerative medicine providing a renewable source of cells for tissue regeneration with potential to form epithelium, neurons, fat tissue (adipocytes) bone and heart muscle (cardiomyocytes), maintaining epigenetic plasticity to maximize progenitor cell differentiation capacity. The enhanced activity of collagen prolyl hydroxylases in the presence of vitamin C can also contribute to higher quality tissues for engraftment, highlighting an important non-epigenetic role of vitamin C in stem cell therapies.

In addition to the challenges facing the safe and effective implementation of stem cell therapies are limitations in the differentiation capacity of iPSCs to generate high quantity and purity of tissues for clinical application (Murry and Keller, 2008; Wu and Hochedlinger, 2011; Scudellari, 2016). Vitamin C, similar to its role in somatic cell reprogramming, has been shown in numerous studies to maintain the proliferation and differentiation potential of ESCs, iPSCs, neural stem cells (NSCs), mesenchymal stem cells (MSCs), epithelial or cardiac progenitors and intestinal stem cells (Figure 2). Importantly, the action of vitamin C in the maintenance of high-quality tissue-specific stem cells has also been attributed to its ability to modulate the activity of aKGDDs, such as TETs and JHDMs, to prevent premature senescence in culture and maintain epigenetic plasticity at tissue-specific gene loci.

Fibroblast-derived iPSCs can be efficiently differentiated into NSCs, giving rise to neuronal and glial cell types in culture that when transplanted into a rat model of Parkinson’s disease (PD) have been shown to improve clinical symptoms (Wernig et al., 2008). PD is characterized by the progressive degeneration of dopamine (DA) neurons in the midbrain (Savitt et al., 2006) and NSCs are considered a potential cell source for the treatment of this disease. In NSC cultures vitamin C treatment protects *in vitro*-expanded NSCs from losing DA neurogenic potential, and promotes DA neuron engraftment by ensuring the faithful expression of midbrain-specific markers in engrafted neurons in rats (Wulansari et al., 2017). Another study also described the role of vitamin C in enhancing NSC differentiation toward DA neurons through boosting of Tet1 and Jmjd3 (Kdm6b) activity (He et al., 2015). Treatment with vitamin C may therefore be important for the maintenance of an epigenetic state that favors enhanced survival of cultured NSCs primed for DA neuron differentiation. These findings mirror the effect of vitamin C in preventing the hypermethylation of germ-line genes in ESC cultures, thus maintaining their differentiation potential toward a blastocyst-like state (Blaschke et al., 2013).

Mesenchymal stem cells directly harvested from adult organs or generated *in vitro* from iPSCs are highly sought after for use in the field of tissue engineering given their potential to differentiate into a variety of cell types including bone (osteoblasts), cartilage (chondrocytes), muscle (myocytes) and fat (adipocytes) (Hynes et al., 2014; Moslem et al., 2015; Soontararak et al., 2018). Growing MSCs as continuous cell sheets in culture as opposed to scaffold-based approaches is believed to more readily mimic the organ tissue microenvironment by preserving cellular junctions and endogenous extracellular matrix (ECM) that results in improved tissue quality and efficacy of engraftment upon transplantation (Yorukoglu et al., 2017).

Vitamin C treatment *in vitro* may also improve the regenerative capacity of stem cells by enhancing collagen synthesis and ECM formation in engineered tissues. Vitamin C plays an essential role in the biosynthesis of collagen from pro-collagen, by enhancing the enzymatic activity of the α-KGDD family of collagen PHs (P4H/P3H) and pro-collagen-lysine α-KG 5-dioxygenases (PLODs) (Knott and Bailey, 1998; Yamauchi and Sricholpech, 2012). In addition to directly enhancing the catalytic activity of collagen PHs, vitamin C also promotes the transcription of collagen genes and increases the stability of collagen mRNA in multiple cell types (Tajima and Pinnell, 1982; Geesin et al., 1991; Qiao et al., 2009; Kim B. et al., 2013). Vitamin C has been shown to up-regulate the expression of extracellular matrix type-I collagen, fibronectin, integrin β1, and promote intercellular junctions that lead to enhanced sheet formation in both *in vivo* animal models and *in vitro* cultured human MSCs (Wei et al., 2012). These studies highlight the non-epigenetic influence of vitamin C supplementation to improve the quality of regenerative tissues for stem cell therapies.

## Preventing Senescence and Aging With Vitamin C

Vitamin C plays an important role in preventing senescence in ESCs and iPSCs during long-term *in vitro* cultures by enhancing the activity of H3K36 demethylases that maintain silencing of the *Ink4/Arf* locus (He et al., 2008; Tzatsos et al., 2009). Given that increased expression of p16/Ink4a is a hallmark of aging (Krishnamurthy et al., 2004) vitamin C could also potentially rejuvenate somatic and adult stem cells by actively silencing expression from this locus. In iPSC-derived cardiomyocytes and MSC cultures vitamin C has also been shown to increase telomerase activity and the expression of genes encoding telomerase-related RNA and protein components that protect telomere stability (Wei et al., 2012; Kim Y.Y. et al., 2013). The regulation of telomere length and the expression of telomerase-related genes that regulate telomerase activity can have a profound influence on cell aging. The ability of vitamin C to suppress senescence and reverse aging phenotypes may be of therapeutic value in the treatment of premature aging.

Patients with Werner Syndrome (WS), exhibit premature aging phenotypes such as gray hair, osteoporosis, diabetes and cancer due to loss-of-function mutations of the WRN gene, which is involved in DNA repair and telomere maintenance (Burtner and Kennedy, 2010; Zhang et al., 2015). Treatment with vitamin C slows down telomere shortening in WS MSCs, and causes down-regulation of p16/Ink4a and decreases the expression of the pro-inflammatory cytokines IL-6 and IL-8 that are associated with a senescence phenotype (Zhang et al., 2013, 2015). Vitamin C-mediated suppression of accelerated aging in WS MSCs is most likely a direct effect of enhanced activity of epigenetic regulators. Increased JHDM activity can suppress expression of the *Ink4/Arf* locus (He et al., 2008; Tzatsos et al., 2009) and TETs are known to play important roles in telomere maintenance, DNA repair and genomic stability (Lu et al., 2014; Kafer et al., 2016; Lazzerini-Denchi and Sfeir, 2016; Yang et al., 2016). Loss of *Tet1* and/or *Tet2* in ESCs causes telomere shortening, chromosomal instability and reduced telomere recombination (Yang et al., 2016). Triple KO of TET1-3 in ESCs has also been shown to cause chromosome segregation defects and increases the frequency of telomere loss and telomere–sister chromatid exchange that may be a compensatory mechanism to counteract telomere shortening (Lu et al., 2014; Kafer et al., 2016; Lazzerini-Denchi and Sfeir, 2016; Yang et al., 2016). Sub-telomeres are hypermethylated in TET depleted ESCs, which may further impede telomere elongation by recombination (Yang et al., 2016). Independently of its catalytic activity, TET2 recruits HDAC2 to selectively repress transcription of *Il6* by enhancing histone de-acetylation at this locus, whereby loss of TET2 leads to increased IL-6 production from innate myeloid cells and increased systemic inflammation (Zhang et al., 2015; Cull et al., 2017). Vitamin C could therefore be considered a rejuvenating epigenetic cofactor, capable of reversing aging phenotypes, such as those observed in patients with WS, and could also be useful in preventing the premature aging of normal differentiated adult cell types.

## Vitamin C-Mediated Regulation of the Cancer Epigenome

Impaired α-KGDD function leads to histone and DNA hypermethylation that is a hallmark of several cancers including hematologic malignancies, such as AML (Figueroa et al., 2010a, b), glioma (Lu et al., 2012; Turcan et al., 2012) and epithelial tumors (Xiao et al., 2012; Killian et al., 2013). Isocitrate dehydrogenases (*IDH1/2)*, are enzymes that generate α-KG making them important players in the regulation of α-KGDD activity and therefore TET and JHDM function. IDH mutations are frequently found in patients with glioma where they are known to drive a DNA hypermethylation phenotype and increase the levels of histone methylation due to impaired TET and JHDM function (Lu et al., 2012; Turcan et al., 2012). In hematologic malignancies, IDH1/2 and TET2 mutations occur in 10–30% of MDS or AML patients and are found to be mutually exclusive, where either can impart an overlapping DNA hypermethylation phenotype (Figueroa et al., 2010a; Akalin et al., 2012; Yamazaki et al., 2015). IDH mutation results in a neomorphic gain of function, where α-KG is consumed by mutant enzymes to produce 2-hydroxyglutarate (2-HG), an oncometabolite that competitively inhibits the catalytic activity of α-KGDDs causing a deficiency in TET (Dang et al., 2009; Ward et al., 2010; Xu W. et al., 2011; Losman et al., 2013) and JHDM function (Lu et al., 2012).

TET proteins are important regulators of DNA methylation fidelity in HSCs, and loss of TET function occurs in a variety of hematologic malignancies (Guillamot et al., 2016; Bowman et al., 2018). A role for vitamin C in regulating TET enzymatic activity to maintain physiological levels of DNA methylation and hydroxymethylation in HSCs therefore has important implications for the prevention and treatment of hematologic malignancy. Based on its role in fibroblast reprogramming it was hypothesized that vitamin C treatment might enhance the enzymatic activity of TET2 to promote 5hmC formation and DNA demethylation in MDS or AML cells (Cimmino et al., 2017). *TET2* mutations in patients are almost always heterozygous (Abdel-Wahab et al., 2009; Delhommeau et al., 2009; Kosmider et al., 2009; Langemeijer et al., 2009), suggesting that enhancing residual TET2 activity, encoded by the remaining wild-type *TET2* allele, or restoring the activity of functionally defective mutant TET2 proteins, could be a viable therapeutic strategy for the treatment of patients with AML. Vitamin C treatment in mouse models of leukemia was shown to mimic genetic restoration of *Tet2* function, causing an increase in 5hmC formation, global DNA hypomethylation, a block in aberrant self-renewal and suppression of disease progression in *Tet2*-deficient mice (Cimmino et al., 2017).

Vitamin C treatment has also been tested on IDH1 mutant mouse leukemia cells where it was shown to induce a Tet2-dependent gain of 5hmC, loss of 5mC and the up-regulation of gene expression signatures that correlate with decreased leukemia stem cell self-renewal and increased differentiation toward a mature myeloid phenotype (Mingay et al., 2018). Mutations in other enzymatic regulators such as succinate dehydrogenase (SDH) and fumarate hydratase (FH) leads to the accumulation of succinate and fumarate, respectively, that also act as oncometabolites by competitively inhibiting TET and JHDM activity even in the presence of sustained α-KG levels (Xiao et al., 2012). The effect of vitamin C treatment in FH and SDH mutant malignancies has not yet been reported, however the responsiveness of *IDH* mutant leukemia cells to vitamin C (despite being depleted of α-KG) suggest that enhancing any residual amount of functional wild-type TET or JHDM activity, even in the presence of inhibitory oncometabolites, could be sufficient to restore epigenetic differentiation cues and erase aberrant DNA and histone hypermethylation phenotypes.

## Vitamin C Deficiency and Cancer Progression

Low plasma vitamin C levels have been reported to be associated with insufficient dietary intake and shorter survival in cancer patients (Anthony and Schorah, 1982; Mayland et al., 2005). Vitamin C deficiency modeled in *Gulo*^–/–^ mice increases the frequency of HSCs in bone marrow and causes a loss of 5hmC in their genome that resembles the effect of TET deficiency (Anthony and Schorah, 1982). Importantly, the suppression of TET activity in HSCs was reversible and could be restored to normal levels upon dietary vitamin C administration (Agathocleous et al., 2017). In this same study, knockout of the vitamin C transporter *Svct2* in bone marrow cells was shown to cooperate with *Flt3*^*ITD*^ oncogene overexpression to accelerate leukemia progression suggesting that cell-intrinsic vitamin C uptake is also an important regulator of cancer (Agathocleous et al., 2017). The majority of patients with hematologic malignancies (up to 58%) are vitamin C deficient compared to normal healthy controls (Huijskens et al., 2016; Liu et al., 2016) and chemotherapy or HSC transplantation can cause vitamin C levels to decrease even further in these patients (Nannya et al., 2014). Even in the absence of TET mutation, vitamin C deficiency in cancer patients may further impair the tumor suppressive function of TET or JHDM proteins, however studies of vitamin C deficiency affecting histone methylation patterns are currently lacking. Given that loss-of-function in *TET2* is known to confer a poor prognosis in AML (Kosmider et al., 2009; Chou et al., 2011), vitamin C deficiency could fuel increased disease aggressiveness or risk of relapse even in non-*TET* mutant cancer patients.

## Vitamin C Treatment – a Novel Targeted Epigenetic Therapy

Epigenetic dysregulation is an important target of interest for therapeutic intervention in the treatment of hematologic malignancies and solid tumors and several recent studies have highlighted the efficacy of vitamin C as a targeted epigenetic therapy. Aberrant DNA methylation patterns are a hallmark of the AML genome, characterized in the majority of AML subtypes by promoter CpG island DNA hypermethylation in the context of global DNA hypomethylation when compared to normal CD34^+^ hematopoietic stem and progenitor cells (Figueroa et al., 2010b; Glass et al., 2017). Inhibitors of DNA methyltransferases (DNMTis), such as 5-azacytidine and decitabine, are approved by the FDA for the treatment of MDS that act by reversing aberrant DNA hypermethylation (Hackanson et al., 2005; Shadduck et al., 2007; Santos et al., 2010). Genome-wide DNA hypo-methylation induced by vitamin C in ESCs is also observed upon treatment of human leukemia cell lines and correlates with increased TET2 activity (Liu et al., 2016; Cimmino et al., 2017). Vitamin C can synergize with decitabine in a TET2-dependent manner to increase 5hmC, drive DNA hypo-methylation and up-regulate the expression of endogenous retroviral genes that triggers an innate viral mimicry response leading to apoptosis of several human cancer cells lines including AML (Liu et al., 2016).

Enhanced TET activity by vitamin C treatment also increases the iterative oxidation of 5hmC to generate 5fC and 5caC, which are recognized by base excision repair (BER) machinery as T:G mismatches in the genome, leading to the recruitment of PARP and GADD45 proteins to facilitate active DNA demethylation (Ciccarone et al., 2012; Shen et al., 2014). Vitamin C treatment in combination with the PARP inhibitor Olaparib, enhances the killing of human AML cells greater than either agent alone (Cimmino et al., 2017), suggesting that TET-mediated DNA oxidation induced by vitamin C can create a targetable synthetic lethality. By forcing AML cells to undergo increased active DNA demethylation, vitamin C treatment renders them hypersensitive to PARP inhibition that can be exploited as a novel combination therapy for the treatment of leukemia.

Vitamin C has also been shown to increase the sensitivity of melanoma cells to bromodomain and extra-terminal inhibitors (BETi) by inducing expression changes associated with a decrease in the level of histone acetylation (Mustafi et al., 2018). In patients with glioblastoma, small pre-clinical and clinical case studies have shown that the combination of conventional treatments with intravenous (i.v.) high-dose vitamin C therapy improved quality of life, progression-free and overall survival (Schoenfeld et al., 2017; Baillie et al., 2018). While the therapeutic mechanism of action in glioma has been attributed to increased intracellular labile-iron toxicity and the prooxidant effects of high-dose vitamin C, an epigenetic response and role for increased TET or JHDM activity in these patients was not investigated.

**FIGURE 3:**
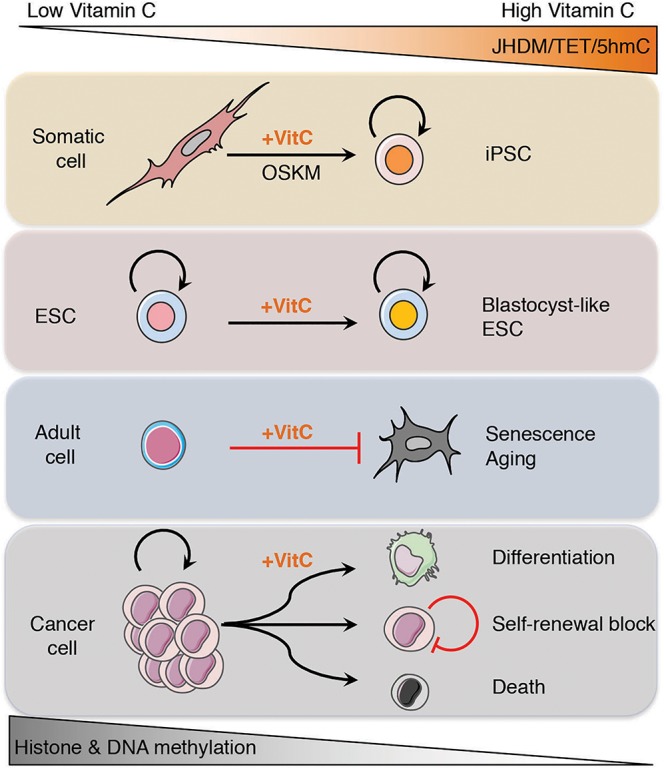
Vitamin C reprograms the epigenome to reverse commitment, prevent senescence and restore differentiation potential. Under growth conditions with low levels of vitamin C, the epigenome of cells becomes hypermethylated, most likely due to suppressed activity of α-ketoglutarate dependent dioxygenases (α-KGDDs) including Jumonji-C domain-containing histone demethylases (JHDMs) and ten-eleven translocation (TET) proteins. By increasing the levels of vitamin C, JHDM, and TET enzymatic activity is enhanced, leading to the loss of histone and DNA methylation, respectively, that promotes somatic cell reprograming, increased differential potential of ESCs toward a blastocyst-like state, protects adult cells from senescence and aging, and can drive the differentiation or death of cancer cells.

Restoring TET function in cancer cells by vitamin C administration, in combination with other targeted epigenetic therapies and hypomethylating agents, may help to erase the epigenetic memory of the cancerous cell state and reprogram the epigenome of these cells that allows them to re-acquire normal differentiation potential and tumor suppressive gene expression programs (Figure 3).

## Dietary Versus Pharmacological Doses of Vitamin C for the Treatment of Cancer

Phase I and II clinical trials are currently in progress to test the efficacy of ascorbate treatment in patients with cancer (Nauman et al., 2018). The relevance of vitamin C as a therapeutic agent will ultimately be dependent on its mode of administration, plasma concentration and capacity for uptake by tumor cells. As little as 10 mg of vitamin C per day prevents the development of scurvy, which is easily obtained with a balanced diet. A recommended daily intake of vitamin C at 200 mg per day is sufficient to maintain an optimal plasma concentration of 70 μM in normal healthy individuals (Lindblad et al., 2013). However, it is estimated that 10% of adults in the United States have marginal vitamin C deficiency, which would equate to <23 μM plasma concentration (Lindblad et al., 2013). Pharmacokinetic studies comparing dietary vs. i.v. administration of high dose vitamin C revealed striking differences in the plasma levels of ascorbate (Mandl et al., 2009). Less than 50% of an oral dose of 1.25 g is absorbed, resulting in maximum peak plasma concentrations of ∼100 μM that fall back to a baseline of ∼70 μM after 4 h (Levine et al., 1996). However, intravenous administration of the same 1.25 g dose of ascorbate can generate a peak plasma level of 1 mM concentration (Padayatty and Levine, 2001; Padayatty et al., 2004). The difference in circulating plasma levels between dietary and i.v. administration have been attributed to the homeostatic down-regulation of the sodium dependent vitamin C transporter 1 (SVCT1) on intestinal epithelial cells in the presence of elevated ascorbate levels (MacDonald et al., 2002). Administration of 1 g of vitamin C in patients with poor renal function has been reported to cause an oxalate build-up that may contribute to renal failure and would therefore not be a viable therapeutic option in these patients (Levine et al., 1996). However, an intermediate dose of vitamin C, above that achieved through oral administration, but not so high to harm kidney function, could be sufficient to treat patients with cancer and act as an adjuvant for existing chemotherapies to enhance their therapeutic potential (Ma et al., 2017; Schoenfeld et al., 2019).

High-dose vitamin C treatment in patients with glioma may also serve as a novel targeted epigenetic therapy in the treatment of this disease. Brain tissue has been reported to have the highest intracellular requirements for vitamin C where it is involved in a variety of pathways within the central nervous system, including the enhancement of norepinephrine biosynthesis, as a cofactor of dopamine β-hydroxylase, and as an inhibitor of glutamate uptake in retinal neurons (Agus et al., 1997; Domith et al., 2018). Vitamin C is able to pass from the blood into the endothelial cells of the blood-brain barrier in its oxidized form, DHA, through glucose transporters (GLUT1) where it can then be reduced back to ascorbate for uptake via SVCT2 that is highly expressed in brain cortex and cerebellar stem cells, neurons and neuroblastoma cells (Agus et al., 1997; Caprile et al., 2009). Given that the blood-brain barrier impedes chemotherapy for gliomas (Zhan and Lu, 2012), the ability of vitamin C to enter the brain may make it a novel and safe treatment option in brain cancers driven by epigenetic dysregulation that could improve the treatment outcome in these patients.

## Conclusion

The ability to reprogram somatic cells into pluripotent stem cells that can then be retrained to differentiate into adult cell lineages has provided us with an invaluable tool for the study and treatment of human diseases. The same mechanisms underlying the erasure of epigenetic memory during reprogramming can also be harnessed in cancer therapy to reverse aberrant epigenetic signatures and allow tumor cells to regain their potential to differentiate or die. The recently described roles of vitamin C as an epigenetic regulator has expanded our understanding of the interplay between the environment and our genome. In addition to improving the quality of stem cells used for regenerative medicine, vitamin C represents a natural, non-toxic, epigenetic therapy that can be used in the prevention and treatment of cancer. Given that loss of function in epigenetic regulators is a hallmark of malignant transformation and a driver of cancer progression, the ability of vitamin C to enhance the activity of epigenetic erasers such as JHDM and TET proteins suggest that future research into the efficacy of vitamin C as an epigenetic therapy should focus on improving the bioavailability and uptake of vitamin C and its general applicability as an adjuvant to existing chemotherapy.

## Author Contributions

TL and EA contributed equally in the writing and organization of the manuscript. LC supervised and edited the manuscript.

## Conflict of Interest Statement

The authors declare that the research was conducted in the absence of any commercial or financial relationships that could be construed as a potential conflict of interest.
